# Loop flexibility in human telomeric quadruplex small-molecule complexes

**DOI:** 10.1093/nar/gkv427

**Published:** 2015-05-04

**Authors:** Gavin W. Collie, Nancy H. Campbell, Stephen Neidle

**Affiliations:** UCL School of Pharmacy, University College London, 29-39 Brunswick Square, London WC1N 1AX, UK

## Abstract

Quadruplex nucleic acids can be formed at the ends of eukaryotic chromosomes. Their formation and stabilisation by appropriate small molecules can be used as a means of inhibiting the telomere maintenance functions of telomerase in human cancer cells. The crystal structures have been determined for a number of complexes between these small molecules and human telomeric DNA and RNA quadruplexes. The detailed structural characteristics of these complexes have been surveyed here and the variations in conformation for the TTA and UUA loops have been explored. Loop conformations have been classified in terms of a number of discrete types and their distribution among the crystal structures. Sugar conformation and backbone angles have also been examined and trends highlighted. One particular loop class has been found to be most prevalent. Implications for in particular, rational drug design, are discussed.

## BACKGROUND—QUADRUPLEX FORM AND FUNCTION

Sequences containing short repetitive G-tracts occur in a number of genomic contexts. They are often able to form higher-order structures, termed quadruplexes (1–3), especially under the influence of appropriate small molecules, proteins or negative supercoiling (4). Historically, the first such sequence category to be so characterised has been in eukaryotic telomeres, which comprise tandem repeats of a G-tract-containing DNA sequence together with associated telomeric proteins. The hexanucleotide repeat in human telomeric DNA is 5′-TTAGGG (5). Almost all of a telomeric DNA sequence (length varies from ∼5 to ∼10 kb) is in duplex form, with the exception of the ∼150–200 nucleotides at the 3′-terminus which are single-stranded (6). This single-stranded DNA (the ‘overhang’) can be folded to form either bimolecular (two-repeat) or unimolecular (four-repeat) quadruplexes, once the associated single-stranded binding proteins (principally several copies of hPOT1), have been competed away (7,8). The concept that quadruplex-binding small molecules can stabilise telomeric quadruplexes (9), has been widely used to discover small molecules with potential anti-cancer activity via inhibition of the action of the telomerase enzyme complex (10–14). Telomerase is a key hallmark of cancer and is highly over-expressed in many cancer cell types whereas it is only expressed at low levels in normal somatic cells, suggesting that it is a plausible anti-cancer target (15,16). The reverse transcriptase action of telomerase catalyses the synthesis of 5′-TTAGGG repeats onto the overhang, to counteract the loss of repeats during replication. This overhang is required to be in a single-stranded form to hybridise with the RNA domain of the telomerase complex and for the catalytic cycle to proceed. Small-molecule induction of the overhang into a four-stranded quadruplex structure, which can be augmented by a quadruplex-binding small molecule (9), results in the overhang becoming inaccessible to the telomerase RNA template, thus effectively inhibiting telomerase catalytic function (17).

## SMALL MOLECULE BINDING TO QUADRUPLEX DNA AND RNA

The demonstration of this concept, that telomerase inhibition can be achieved via quadruplex stabilisation with anthraquinone derivatives (9,18) and subsequently with many other small molecules (10–13) has led to the widespread development of this approach to selectively targeting human cancers. Information on the large and structurally diverse superfamily of quadruplex-binding small molecules is available from the G4LDB database www.g4ldb.org (19).

Several compounds including the acridine derivatives BRACO-19 (20), RHPS4 (21), the cyclic natural product telomestatin (22) and its analogues, and some tetra-substituted naphthalene diimide (ND) derivatives (23), have been shown to selectively affect telomere maintenance in cancer cells. Mechanistic studies have demonstrated that the cellular responses to these agents, which occur considerably more rapidly than would be expected on the basis of a classic telomerase inhibition and telomere attrition model, involve the activation of a DNA damage response, presumably by sensing the presence of a quadruplex nucleic acid signal which is not associated with and protected by, telomeric proteins (20,21,24–30). This response is an important factor in the rapidity and selectivity of the growth-inhibitory action of these quadruplex-binding ligands in tumour cells and tumour xenografts (19,20,24).

The frequency of occurrence of quadruplex-forming sequences other than at telomeres in the human genome has been evaluated by informatics approaches (31–33). These studies have shown that such sequences are especially prevalent in promoter sequences (34) and in 5′-UTRs (35–37). Their over-representation in a number of oncogene promoter sequences (33) has led to the hypothesis that promoter quadruplexes can be targets for therapeutic intervention using small molecules which stabilise a particular quadruplex structure within a promoter sequence (38,39). This in principle results in inhibition of the transcription of that particular gene. The concept has been evaluated with small molecules targeting in particular the *c-MYC* and *c-KIT* promoter quadruplexes (see for example 10–13,39), although the challenge remains of devising small molecules capable of selectively targeting a particular quadruplex in the absence of off-target effects on other quadruplexes. Importantly, the concept of quadruplexes as targets in human cancer has been validated by their direct visualisation in human cells and tissues (40–42).

Extensive structural data on native human telomeric quadruplexes (HTQs) are now available from crystallographic and nuclear magnetic resonance (NMR) studies (43–53), whereas there is currently structural information available on only a small number of non-telomeric quadruplexes, of which the c-*KIT*, c-*MYC* and *BCL-2* promoter quadruplexes are the best-studied (54–64). Detailed NMR structures have been determined for three small-molecule complexes with c-*MYC* quadruplexes: bound to the porphyrin ligand TMPyP4 (PDB ID 2A5R) (65), bound to the bis-quinolinium compound Phen-DC(3) (PDB ID 2MGN) (66) and with a drug-like quindoline compound (PDB ID 2L7V) (67).

The available structural data on human telomeric quadruplex (HTQ) complexes with small molecules are dominated by results from X-ray crystallographic studies, with 16 structures deposited to date in the PDB (68–76). Of the few NMR studies on human telomeric quadruplex-small molecule complexes that have been reported, only one is of an intramolecular complex, bound to a derivative of the drug telomestatin (PDB ID 2MB3) (77). The present study focusses on the crystal structures and in particular, analyses aspects of their conformational variability in order to establish unifying trends and features. There are currently no crystal structures available for small-molecule complexes with promoter or 5′-UTR quadruplexes and only a very few NMR structures (involving promoter quadruplexes), but some of the underlying principles outlined below will also apply to these complexes. One of the small-molecule complexes in the database is with a bimolecular human telomeric RNA (TERRA) quadruplex (71), and this is complemented by the availability of a crystal structure of the native RNA quadruplex (78).

**Figure 1.**
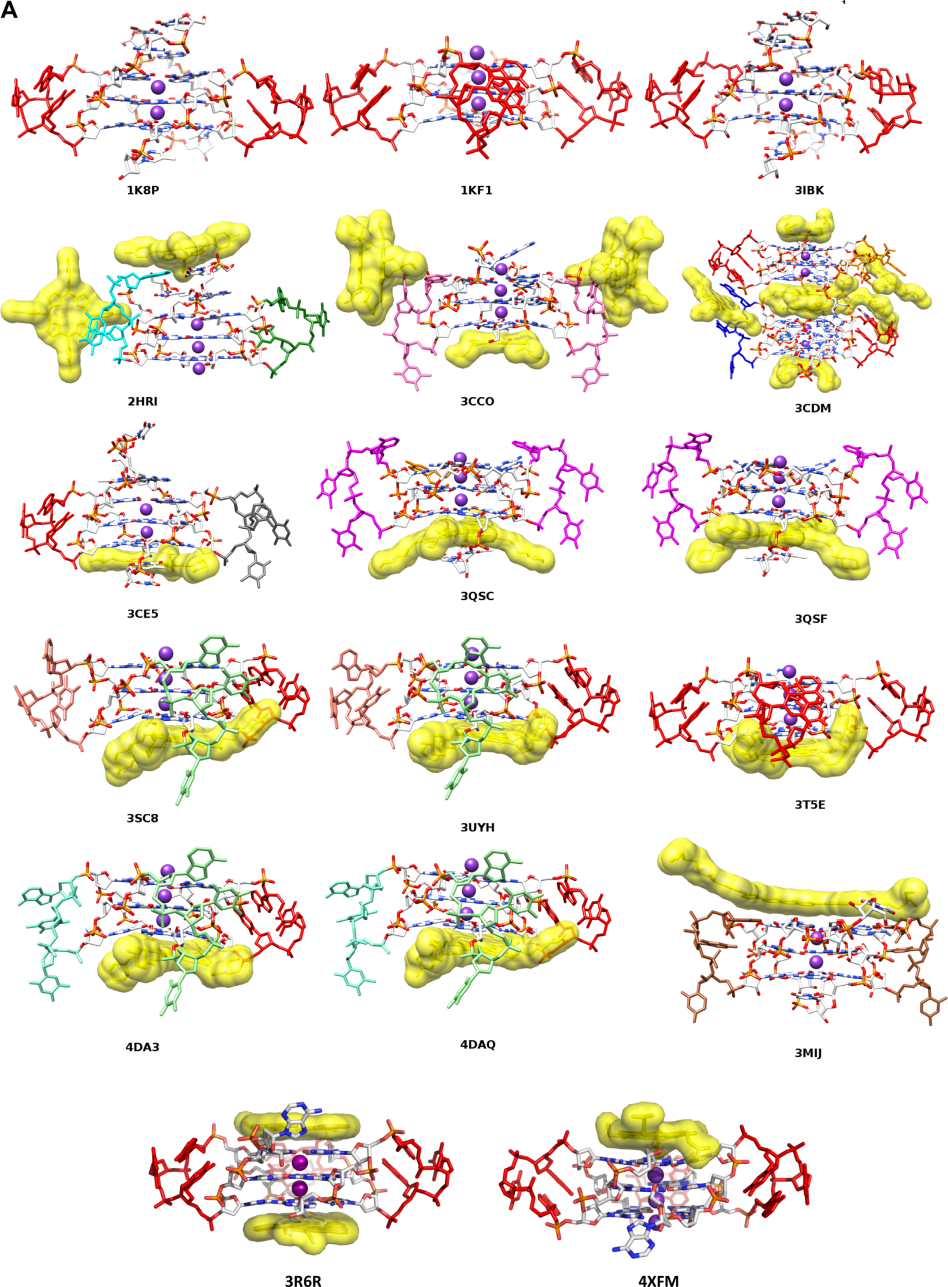

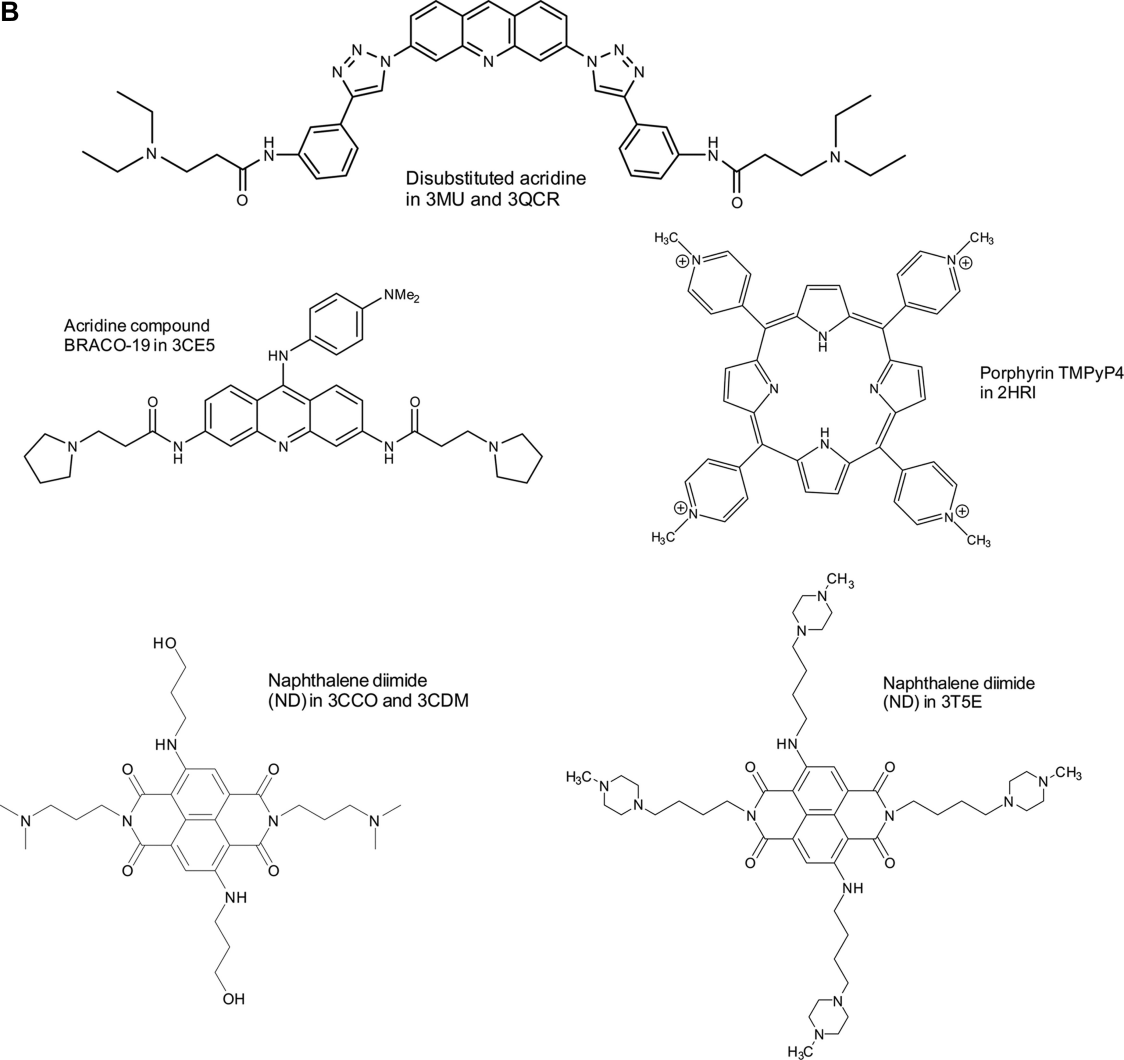

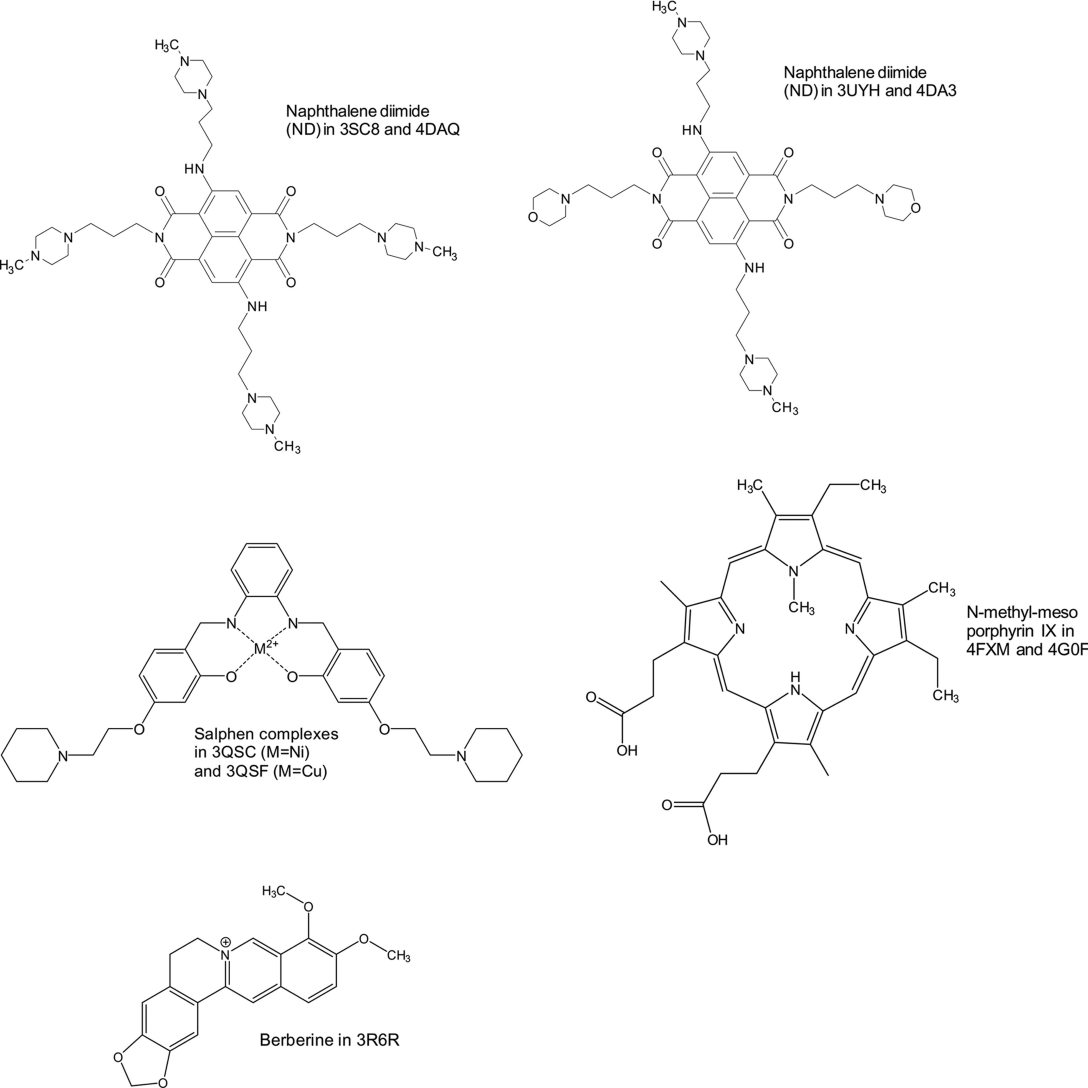
(**A**) View of the human telomeric quadruplex native and ligand complex crystal structures, with nucleic acids drawn in stick form and ligands in surface representation. Metal ions (potassiums) are shown as purple spheres. Individual loops are highlighted with sticks in single colours. The colour coding corresponds to the more detailed view of each loop type shown in Figure 2. (**B**) Structures of individual ligands, shown with the PDB IDs for the structures in which they occur.

The overwhelming majority of quadruplex-binding small molecules reported to date possess planar groupings and their mode of binding predominantly involves 3′ or 5′ end-stacking onto terminal G-quartets (Figure 1). Intercalative binding has not been observed experimentally for these ligands and is widely considered not to be consistent with biophysical data on quadruplex-small molecule complexes (11–13). A small number of quadruplex-binding ligands, mostly based on a polyamide motif, have been proposed to bind in quadruplex grooves rather than on G-quartet surfaces. There is no detailed experimental structural information on their interaction with human telomeric bimolecular or unimolecular quadruplexes and so groove-binding structures will not be considered any further here.

As a consequence of the rise of the quadruplex targeting concept, a large number of diverse quadruplex-binding small molecules have been devised and evaluated. Most of these have been discovered by biophysical screens of compound libraries (11–13). Rather fewer studies have employed rational design and optimisation of ligands using structural data from X-ray crystallography and NMR studies. *In silico* docking methods, mostly using native quadruplex structures as starting-points, have been used in a few instances to screen large compound libraries, (79–81). Experimental structures are crucial starting-points for *in silico* studies so it is hoped that the analyses and correlations presented here will help provide sound starting-points for such future studies.

## THE FLEXIBILITY OF HUMAN TELOMERIC QUADRUPLEXES

It is now well-established that intramolecular quadruplexes comprising four repeats of the human telomeric sequence 5′-TTAGGG, can form a variety of topological isomers in solution, differing in the orientation of the backbone and the nature of the loops formed by the ‘spacer’ TTA triplet sequences (which can adopt lateral, diagonal or parallel arrangements dependent on the orientation of the attached strand). A number of these quadruplex topological isomers have been identified by NMR methods (43–50); the parallel form with all loops of the propeller type (also termed strand-reversal) is the sole form found in the X-ray crystallographic studies (52). The observation of a particular quadruplex topological isomer in solution is dependent on a number of factors, primarily quadruplex concentration, the presence or otherwise of a molecular crowding environment, the nature of the counter-ion and the flanking sequences at the 5′ and 3′ ends (see for example 51,82–85). There continues to be controversy as to which topological isomer is the most biologically relevant, although it is plausible that both parallel and anti-parallel ones can co-exist in the high local concentration of cellular conditions. Small molecule binding can induce the stabilisation of a particular form (for example the induction of the parallel topology by N-methylmesoporphyrin IX (86)); although there is to date very incomplete understanding of the molecular basis for the preference for a particular topology; accessible surface area of a terminal G-quartet has been suggested as being an important factor (87).

This diversity in native HTQ topologies has not been reflected in the folds observed in the crystal structures of small-molecule complexes with HTQs. All have been found to have a parallel topology, regardless of whether they are bimolecular or unimolecular complexes (Table 1). They crystallise in ten different crystallographic space groups, strongly suggesting that crystal packing forces are not the determinant of the single observed (parallel) topology. The present survey has examined in particular the principal nucleic acid conformational variable regions in these structures, the TTA loops. The structural cores of all quadruplexes comprise two or more stacked G-quartets. In the case of HTQ crystal structures the core comprises three stacked G-quartets, which has been observed to be exceptionally stable. Structural alignment of the three stacked G-quartets of all HTQ crystal structures analysed in this work using the native HTQ structure (PDB ID 1KF1) as a reference gives an average r.m.s.d of just 0.86 Å (see Supplementary Table S1 and Figure S1). This stability of the HTQ G-quartets has also been confirmed by molecular dynamics simulations (88–91). Accordingly, the geometry of the G-quartet core has not been surveyed here.

**Table 1.** Native DNA and RNA human telomeric quadruplexes and small-molecule complex crystal structures showing PDB codes, basic crystallographic data and TTA/UUA loop types

PDB ID	Sequence	Description of structure	Resolution (Å)	Space group	R-free	R-factor	No. of crystallographically unique loops	Loop type	Ref.
1K8P	d[U_Br_AG_3_U_Br_TAG_3_T]	Native DNA	2.40	P3_1_2 1	0.280	0.193	2^a^	Type-1	(52)
1KF1	d[AG_3_(T_2_AG_3_)_3_]	Native DNA	2.10	P6	0.263	0.231	3	Type-1 (x3)	(52)
2HRI	d[TAG_3_T_2_AG_3_]	Porphyrin TMPyP4 complex	2.09	C222_1_	0.257	0.208	2	Type-2	(68)
								Type-3	
3CE5	d[TAG_3_T_2_AG_3_T]	Acridine complex	2.50	I4	0.213	0.184	2	Type-1	(70)
								Type-7	
3CCO	d[TAG_3_T_2_AG_3_T]	ND^b^ complex	2.20	P6_4_22	0.303	0.257	1	Type-4	(69)
3CDM	d[TAG_3_(T_2_AG_3_)_3_]	ND complex	2.10	P2_1_	0.295	0.234	6	Type-1 (x4)	(69)
								Type-5	
								Type-6	
3IBK	r(U_Br_AG_3_U_2_AG_3_U)	Native RNA	2.20	P3_1_21	0.231	0.216	2^c^	Type-1	(78)
3MIJ	r(UAG_3_U_2_AG_3_U)	RNA acridine complex	2.60	P23	0.248	0.236	1	Type-8	(71)
3QCR	d[TAG_3_T_2_AG_3_T]	Acridine complex.	3.20	P6_2_22	0.378	0.346	1^d^	NA	(71)
3SC8	d[AG_3_(T_2_AG_3_)_3_]	ND complex	2.30	P3_1_21	0.288	0.260	3	Type-1	(72)
								Type-10	
								Type-11	
3T5E	d[AG_3_(T_2_AG_3_)_3_]	ND complex	2.10	P6	0.282	0.245	3	Type-1 (x3)	(72)
3QSC	d[AG_3_T_2_AG_3_T_2_]	Salphen complex	2.40	C222	0.234	0.214	1	Type-9	(75)
3QSF	d[AG_3_T_2_AG_3_T_2_]	Salphen complex	2.40	C222	0.320	0.240	1	Type-9	(75)
3UYH	d[AG_3_(T_2_AG_3_)_3_]	ND complex	1.95	P3_1_21	0.275	0.231	3	Type-1	(76)
								Type-10	
								Type-11	
4DA3	d[G_3_(T_2_AG_3_)_3_]	ND complex	2.40	P3_1_21	0.282	0.242	3	Type-1	(76)
								Type-10	
								Type-12	
4DAQ	d[G_3_(T_2_AG_3_)_3_]	ND complex	2.75	P3_1_21	0.275	0.212	3	Type-1	(76)
								Type-10	
								Type-12	
3R6R	d[AG_3_(T_2_AG_3_)_3_]	Berberine complex	2.30	P6	0.252	0.217	3	Type-1 (x3)	(73)
4G0F	d[AG_3_(T_2_AG_3_)_3_]	Mesoporphyrin complex	2.15	P6	0.262	0.239	3	Type-1 (x3)	(74)
4FXM	d[AG_3_(T_2_AG_3_)_3_]	Mesoporphyrin complex	1.65	P2_1_2_1_2	0.262	0.224	3	Type-1 (x3)	(74)
Total no. of experimentally determined TTA or UUA loops: 43									
Total no. of distinct TTA loop types: 12									
Total no. of type-1 TTA loops occurrences: 26									
Total number of crystal structures: 19									
Total no. of distinct space groups: 10									
Total no. of HTQ-ligand complexes: 16									

^a+c^Chain B loop not resolved in the electron density; ^b^ND, Naphthalene diimide; ^d^Loop poorly resolved in the electron density.

### Methodology used in the survey

Atomic coordinates for the telomeric quadruplex structures were extracted from the Protein Data Bank (www.rcsb.org), with geometric and conformational analyses performed using the 3DNA program (92) at www.x3dna.org and visualised with the programs CHIMERA (93) at www.cgl.ucsf.edu/chimera and PyMOL (94) at www.pymol.org. Each loop was analysed individually and no averaging of conformational features was done. Disordered and poorly resolved loops were excluded from the analyses. Sugar puckers were assigned directly from pseudorotation parameters calculated by 3DNA from crystallographic coordinates.

## STRUCTURAL ANALYSIS AND BACKBONE DIHEDRAL ANGLE COMPARISON OF HTQ TTA LOOPS

**Figure 2.**
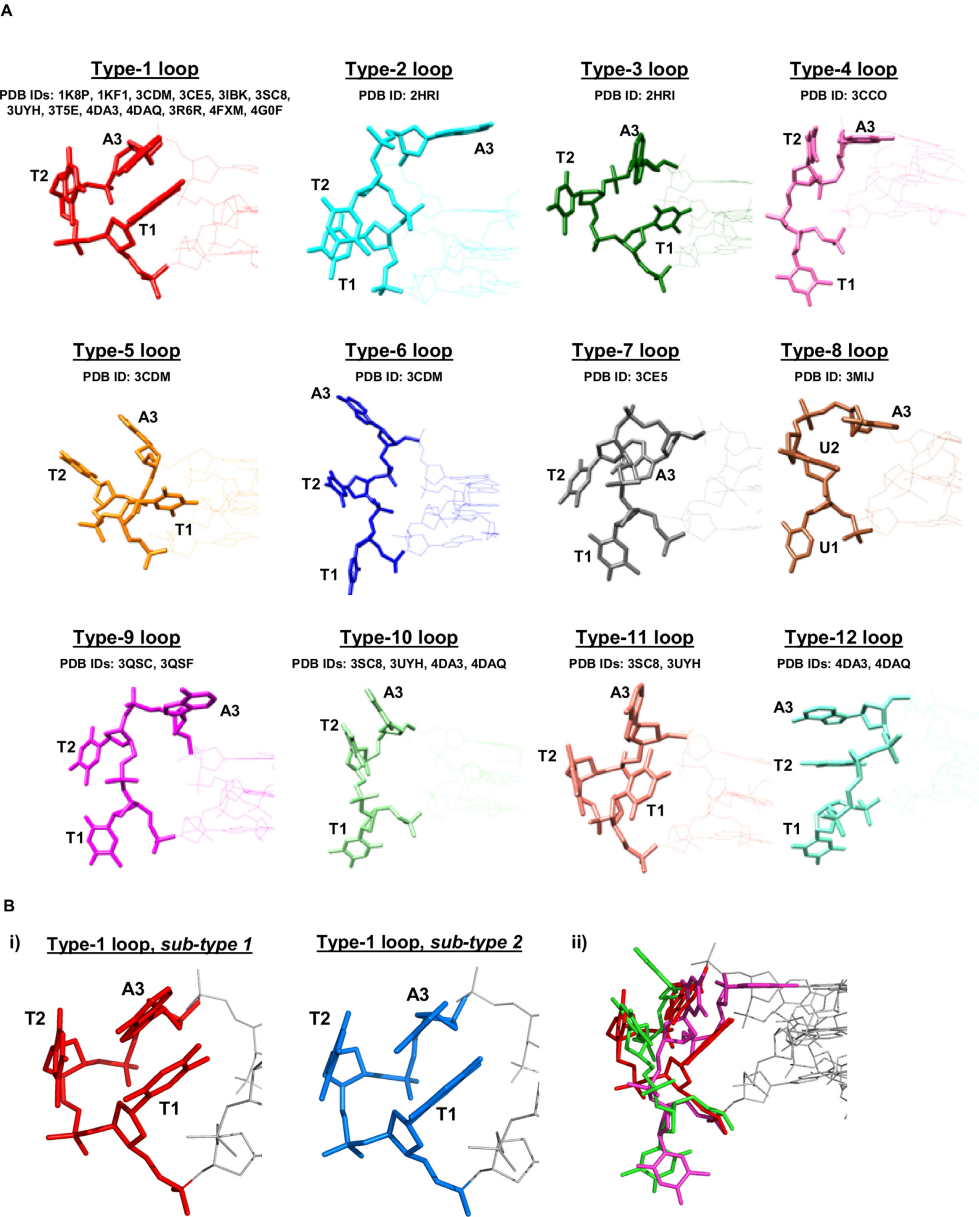
(**A**) Views of the various TTA loop types (coloured as in Figure 1), grouped into 12 distinct categories. (**B**) (i) The two sub-types of type-1 loops. Sub-type 1 (left) is from PDB structure 1KF1, sub-type 2 (right) is from PDB structure 3T5E. The structures shown here have been structurally aligned and are presented in the same orientation. (ii) An example of the structural diversity of non-type-1 loops: overlay of a type-4 loop (pink, from PDB structure 3CCO), a type-10 loop (green, from PDB structure 3SC8) and a type-1 loop (red, from PDB structure 1KF1). The non-type-1 loops are dramatically different to the ‘native’ type-1 TTA loop—as well as to one another. Note the retention of base-stacking interactions in the type-10 loop arrangement, which is a common feature of non-type-1 loops.

There are currently 19 X-ray crystal structures of native and small-molecule-complexed human telomeric DNA and RNA G-quadruplexes available in the Protein Data Bank. All of these contain d(TTA) (or r(UUA)) propeller loops (Figure 1a, Table 1). This data set comprises 17 DNA and two RNA (i.e. TERRA/telRNA) structures. The structures of individual ligands are shown in Figure 1b. These bimolecular and unimolecular quadruplex native and ligand-bound structures represent a total of 43 crystallographically-determined propeller-type loops (this number excludes three poorly-resolved loops: see Table 1). The range of resolution in these analyses is 1.65–3.2 Å, with an average of 2.30 Å. The loops have been categorised into 12 distinct groups (termed type-1, type-2…etc) on the basis of their overall three-dimensional shape (see Table 2 and Figure 2a, b).

**Table 2.** Qualitative TTA/UUA loop descriptors in terms of base stacking

Loop type	Stacked bases	Mutually perpendicular bases	Orientation of 3^rd^ base
1	T1, A3	T2, A3	T2 close to A3
2	T1, T2	-	A3 away from T1, T2
3	T2, A3	T1, A3	T1 close to A3
4	-	T2, A3	T1 away from T2, A3
5	T2, A3	-	T1 close to T2, A3
6	T2, A3	T1	T1 away from T2, A3
7	T2, A3		T1 close to T2
8	U2, A3	U1	U1 away from U2, A3
9	No stacking	-	All away from each other
10	T2, A3	-	T1 away from T2, A3
11	No stacking	T1, T2	A3 close to T1
12	T2, A3	-	T1 away from T2, A3

### Type-1 propeller loops

**Figure 3.**
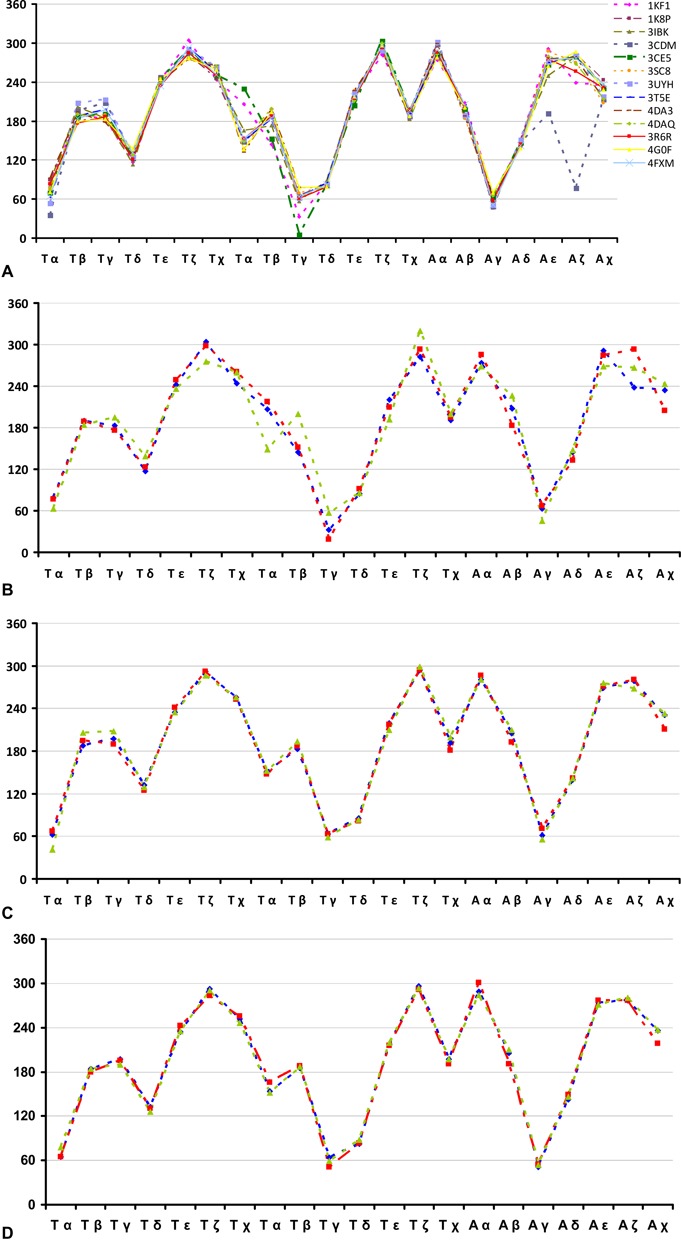

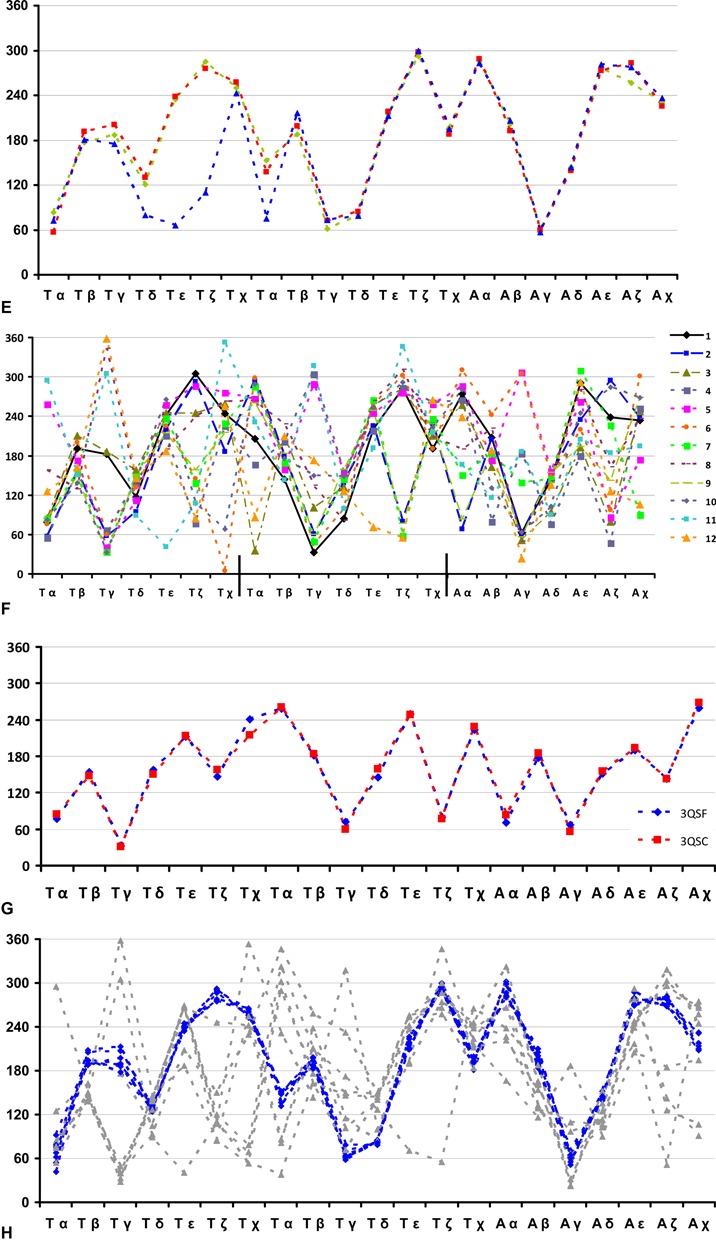

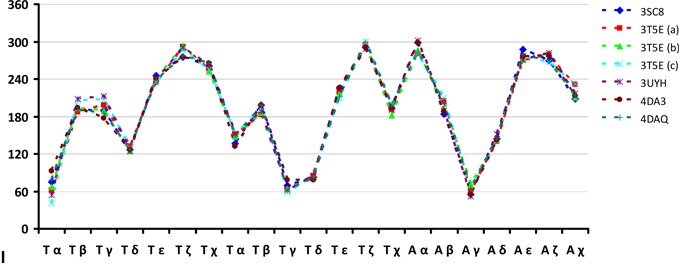
Plots of nucleotide conformational angles in the TTA loops. (**A**) *Backbone and glycosidic torsion angles*. These are shown for all structures containing at least one type-1 TTA (or UUA) loop. For structures which contain more than one type-1 loop only the first TTA loop is represented. (**B**) *Comparison of backbone and glycosidic torsions angles*. These are shown of the three type-1 propeller loops of the native human telomeric unimolecular quadruplex (PDB ID 1KF1) (each loop coloured separately; red, green or blue). (**C**) *Comparison of backbone and glycosidic torsions angles*. These are for the three type-1 propeller loops of a naphthalene diimide telomeric quadruplex complex (PDB ID 3T5E) (each loop coloured separately; red, green or blue). (**D**) *Comparison of the backbone and glycosidic torsions angles*. These are for the three type-1 propeller loops of the mesoporphyrin complex with a human telomeric quadruplex (PDB ID 4FXM) (each loop coloured separately; red, green or blue). (**E**) *Comparison of the backbone and glycosidic torsions angles*. These are for the three type-1 propeller loops in the berberine complex with a human telomeric quadruplex (PDB ID 3R6R) (each loop coloured separately; red, green or blue). (**F**) *Dihedral angle plots for each of the TTA residues of each of the 12 loop types* (each trace represents a distinct loop type). The loop 1 of 1KF1 (solid black line) can be used as a common reference. Dihedral angles are derived from loops from PDB structures 1KF1 (1), 2HRI (2 and 3), 3CCO (4), 3CDM (5 and 6), 3CE5 (7), 3MIJ (8), 3QSC (9), 3SC8 (10 and 11) and 4DA3 (12). (**G**) *Type-9 loop backbone and glycosidic dihedral angles*. These are from bimolecular human quadruplexes (PDB IDs 3QSF and 3QSC) complexed with salphen ligands. (**H**) *Comparison of backbone and glycosidic dihedral angles in the TTA loops of the naphthalene diimide (ND) complex structures*. Type-1 TTA loops are shown as blue dashed lines. Non-type-1 loops are shown as grey dashed lines. Structures represented are: PDB IDs 3SC8, 3T5E, 3UYH, 4DA3 and 4DAQ. (**I**) *Comparison of TTA loop backbone and glycosidic dihedrals of naphthalene diimide complex crystal structures, for type-1 loops only*.

By far the most commonly observed loop type is the intercalated ‘TAT’ loop found in the native telomeric DNA structures (PDB IDs 1KF1 and 1K8P), termed type-1 loops. This loop arrangement always involves the adenine residue π-π stacking above the first thymine, with the second (central) thymine adopting one of two similar orientations: either positioned perpendicular to the G-quartet planes or stacked (or nearly stacked) on the external face of the adenine (Figure 2b (i)). Type-1 loops have been found to occur in 13 crystal structures to date (including both native and ligand-complexes), with a total of 26 occurrences overall (therefore comprising over 60% of all crystallographically-observed TTA loops). This strongly suggests that the type-1 propeller loop is an energetically favourable and stable arrangement, which is independent of crystal packing mode—Table 1 also shows that these structures occur in a variety of crystallographic space groups (five distinct space groups in total). It is perhaps significant that, although ligand binding events often result in significant rearrangement of the type-1 loop geometry (described below), ten distinct quadruplex-ligand complexes exist in which the type-1 loop is preserved. This implies further that the type-1 loop arrangement is a structurally robust motif. Comparison of the backbone and glycosidic dihedral angles for the type-1 loops of each of the 13 structures which contain such a loop reveals a high degree of conservation in backbone geometry (Figure 3a). The only significant deviation can be seen for a 23-mer-naphthalene diimide derivative complex structure (PDB ID 3CDM), which has changes in the ε and ζ angles for the loop adenine residue (Figure 3a).

Backbone dihedral comparison for the structures which contain multiple type-1 loops reveals a similar level of angle conservation. For example, the native 22-mer structure (PDB ID 1KF1) and the naphthalene diimide derivative-complexed 22-mer (PDB ID 3T5E, with identical sequence) both contain three type-1 loops, which display a significant level of intramolecular correlation when comparing backbone dihedrals (Figure 3b, c, respectively). A similar level of correlation in dihedral angles is also seen within the 22-mer complexes involving mesoporphyrin (PDB ID 4FXM) and berberine (PDB ID 3R6R) (Figure 3d, e, respectively).

### Non type-1 propeller loops

Of the 43 crystallographically observed TTA (or UUA) propeller loops, less than 40% (17 in total) adopt conformations other than the intercalated type-1 loop arrangement described above. These non-type-1 loops adopt a wide variety of dissimilar and diverse structures in the crystal state, which can be divided into 11 distinct groups (type-2 to type-12) based on the orientation of the three loop bases (Figure 2a and Tables 1 and 2). The structural diversity of the 11 non-type-1 loops indicates that TTA propeller loops possess a significant degree of structural flexibility, however, this does not necessarily signify that the crystallographically observed loop types are unstable. Indeed, the majority of these non-type-1 loop arrangements share the common feature of exploiting intra-loop base π-stacking interactions (Figure 2a and b(ii) and Table 2)—a feature which would be expected to confer a degree of stability upon these loop structures.

Comparison of backbone and glycosidic dihedral angles of the 11 non-type-1 loops reveals a broad distribution in backbone geometry for the different loop arrangements (Figure 3f), which is to be expected, considering the dramatic structural variety of non-type-1 loops (Figure 2). However, there do appear to be several favourable groupings of torsion angles—such as a preference for *anti* glycosidic bond angles, particularly for the second loop thymine—in addition to some correlation with the type-1 backbone dihedral angle distribution. As with type-1 loops, backbone dihedral angle comparison of equivalent loop types between different structures reveals a high level of similarity, as seen when comparing the type-9 loops observed in the two bimolecular salphen-complexed quadruplexes 3QSF and 3QSC (Figure 3g).

Although type-1 loops are indeed observed in a number of HTQ-ligand complexes (as stated above), all non-type-1 loops occur in ligand-bound HTQ structures, thus, non-type-1 loops are almost certainly a consequence of ligand-binding events. If one considers the type-1 loop to represent the ‘native’ TTA loop conformation, the observation of a further 11 distinct loop conformations—many of which are dramatically different to the type-1 arrangement (see for example Figure 2b(ii))—indicates clearly that TTA propeller loops of human telomeric G-quadruplexes possess a significant degree of structural polymorphism. This observation would be expected to have important consequences with respect to the rational design of small molecules targeted towards HTQs, however, the almost paradoxical observation of both type-1 and non-type-1 loops within a single ligand-bound HTQ structure (e.g. PDB IDs 3CDM, 3CE5, 3SC8, 3UYH, 4DA3, 4DAQ) precludes a clear conclusion: small molecule compounds are able to elicit both dramatic and negligible effects on TTA loop geometry.

## SUGAR PUCKER ANALYSIS AND COMPARISON OF HTQ TTA LOOPS

### Sugar pucker distribution of type-1 propeller loops

Comparison of the deoxyfuranose and furanose sugar puckers in the nucleotides of all type-1 propeller loops reveals an exceptionally high degree of similarity in pucker distribution. Of the 26 type-1 loops observed in crystal structures, 17 (65.4%) adopt a sugar pucker distribution of C1’-*exo*, C3’-*endo*, C2’-*endo* respectively for the three T-T-A nucleotides in a loop (Table 3). Interestingly, this consensus pattern of pucker arrangement—a similar pucker range to those found in DNA duplex crystal structures—is also observed for RNA G-quadruplex type-1 loops. Analysis of the frequency of pucker-types for each loop residue (i.e. T, T, A/U, U, A) reveals a high level of correspondence, with the first thymine sugar adopting a C1’-*exo* pucker in 69.2% of observed instances, the second thymine adopting a C3’-*endo* pucker conformation in 100% of the observed instances and the adenine sugar adopting a C2’-*endo* pucker in 88.5% of all type-1 loops (Table 4). The high level of correlation in the distribution of sugar pucker geometry and backbone dihedral angles of type-1 loops strongly implies that the folding of TTA triplets into the type-1 arrangement is a highly precise, non-random process.

**Table 3.** Pucker distributions for TTA/UUAs of all type-1 loops

	**1KF1-a**	**1KF1-b**	**1KF1-c**	**1K8P**	**3IBK**	**3CDM-a**	**3CDM-b**	**3CDM-c**	**3CDM-d**	**3CE5**	**3SC8**	**3UYH**	**3T5E-a**
T	C1’-*exo*	C1’-*exo*	C1’-*exo*	C1’-*exo*	C1’-*exo*	C1’-*exo*	C4’-*exo*	C1’-*exo*	C1’-*exo*	C1’-*exo*	C1’-*exo*	C1’-*exo*	C2’-*endo*
T	C3’-*endo*	C3’-*endo*	C3’-*endo*	C3’-*endo*	C3’-*endo*	C3’-*endo*	C3’-*endo*	C3’-*endo*	C3’-*endo*	C3’-*endo*	C3’-*endo*	C3’-*endo*	C3’-*endo*
A	C2’-*endo*	C2’-*endo*	C2’-*endo*	C2’-*endo*	C2’-*endo*	C2’-*endo*	C2’-*endo*	C2’-*endo*	C1’-*exo*	C2’-*endo*	C2’-*endo*	C2’-*endo*	C2’-*endo*
	**3T5E-b**	**3T5E-c**	**4DA3**	**4DAQ**	**3R6R-a**	**3R6R-b**	**3R6R-c**	**4FXM-a**	**4FXM-b**	**4FXM-c**	**4G0F-a**	**4G0F-b**	**4G0F-c**
T	C1’-*exo*	C2’-*endo*	C1’-*exo*	C1’-*exo*	C1’-*exo*	C1’-*exo*	C4’-*exo*	C2’-*endo*	C1’-*exo*	C1’-*exo*	C2’-*endo*	C2’-*endo*	C2’-*endo*
T	C3’-*endo*	C3’-*endo*	C3’-*endo*	C3’-*endo*	C3’-*endo*	C3’-*endo*	C3’-*endo*	C3’-*endo*	C3’-*endo*	C3’-*endo*	C3’-*endo*	C3’-*endo*	C3’-*endo*
A	C2’-*endo*	C2’-*endo*	C2’-*endo*	C2’-*endo*	C2’-*endo*	C2’-*endo*	C2’-*endo*	C2’-*endo*	C2’-*endo*	C2’-*endo*	C1’-*exo*	C1’-*exo*	C2’-*endo*

Yellow indicates a sugar which deviates from the consensus pucker conformation. Lower-case letters denote crystallographically unique type-1 loops within a single crystal structure.

**Table 4.** Percentage of each residue in type-1 loops which adopt sugar pucker consensus ‘C1’-*exo*, C3’-*endo*, C2’-*endo*’

	Pucker type	% Observed
T	18/26 C1’-*exo*	69.2%
T	26/26 C3’-*endo*	100.0%
A	23/26 C2’-*endo*	88.5%

C3’-*endo* pucker for the second loop thymine is 100% conserved.

### Sugar pucker distribution of non-type-1 propeller loops

As with backbone dihedral angle distribution, the sugar puckering of non-type-1 loops is also highly variable when comparing all 11 non-type-1 loops (Table 5). Although there is no common consensus pucker arrangement for the non-type-1 loops, there is a clear shift from the ‘C1*’-exo*, C3’*-endo*, C2’*-endo*’ consensus of type-1 loops to a C2’*-endo* conformation for *all* loop residues. For example, the first thymine of the TTA loop adopts a C2’-*endo* pucker in 52.9% of all non-type-1 propeller loops, compared to 23.1% in all type-1 loops (6/26 occurrences) (Table 6). This presumably reflects the energetic preference for C2’-*endo* puckered sugars in DNA, and suggests the less favourable C1’-*exo* and C3’-*endo* of the first two residues of the type-1 loops may be compensated for by the favourable energetics of base-base π-stacking interactions.

**Table 5.** Sugar pucker distributions for each non-type-1 loop

	2RHI 2*	2RHI 3	3CCO 4	3CDM 5	3CDM 6	3CE5 7	3MIJ 8	3QSC 9	3QSF 9	3SC8 10	3SC8 11	3UYH 10	3UYH 11	4DA3 10	4DA3 12	4DAQ 10	4DAQ 12
T	C3’-*endo*	C2’-*endo*	C2’-*endo*	C1’-*endo*	C2’-*endo*	C2’-*endo*	C3’-*exo*	C2’-*endo*	C2’-*endo*	C2’-*endo*	C3’-*endo*	C2’-*endo*	O4’-*endo*	C2’-*endo*	C3’-*exo*	C1’-*exo*	C4’- *exo*
T	C1’-*exo*	C1’-*exo*	C2’-*endo*	C2’-*endo*	C3’-*endo*	C2’-*endo*	C3’-*endo*	C2’-*endo*	C2’-*endo*	C3’-*exo*	C2’-*exo*	C2’-*endo*	C3’-*endo*	C2’-*endo*	C2’-*endo*	C2’-*endo*	C2’- *endo*
A	C2’-*endo*	O4’-*endo*	C4’-*exo*	C2’-*endo*	C2’-*endo*	C2’-*endo*	C2’-*endo*	C2’-*endo*	C2’-*endo*	C1’-*exo*	C4’-*exo*	C1’-*exo*	C2’-*endo*	C1’-*exo*	C1’-*exo*	C1’-*exo*	C1’- *exo*

*Numbers below PDB codes denote loop type.

**Table 6.** Occurrence of each pucker conformation in non-type-1 loops

	C1’-*endo*	C2’-*endo*	C3’-*endo*	C1’-*exo*	C2’-*exo*	C3’-*exo*	C4’-*exo*	O4’-*endo*	% C2’-*endo* for non-type-1 loops	% C2’-*endo* for type-1 loops
**T**	1	**9**	2	1	0	2	1	1	52.9% (9/17)	23.1% (6/26)
**T**	0	**10**	3	2	1	1	0	0	58.8% (10/17)	0.0% (0/26)
**A**	0	**8**	0	6	0	0	2	1	47.1% (8/17)	88.5% (23/26)

There is a pronounced shift towards *C2’-endo* puckering for all residues of the TTA loops for non-type-1 loops when compared to type-1 loops (highlighted in bold).

## ANALYSIS AND CONCLUSIONS

The solution NMR evidence for type-1 loops, which together with the diversity of crystal packing modes in the crystal structures, suggests that crystal packing forces are not contributing to the loop types. The diversity of space groups and resulting patterns of molecular packing in these crystals suggests that the intermolecular packing forces are weak and therefore different packing arrangements can readily occur. This is consistent with the assumption that what is observed in the crystalline state are low-energy conformations of these complexes. We suggest that here, the preferred conformations of the relatively flexible loops are always among those most observed in these crystal structures.

Although the type-1 loop is present in five distinct space groups, many of the non-type-1 loops are observed less frequently, and thus there is not currently sufficient data to make any definitive statements about the relationship between space group and loop type. It should be noted, however, that the relationship between space group and (loop) conformation is not necessarily a one way process—a particular space group may affect the loop geometry, but equally, a loop type can influence the space group into which the molecule/DNA/RNA packs, i.e. loop-type may define the crystal packing, rather than the packing defining the loop-type.

Although there is no direct evidence that non-type-1 loops are produced by ligand binding, it is clear that ligand binding is involved in some way in the formation of non-type-1 loop arrangements. Since type-1 loops are also observed in the native DNA and RNA structures, we suggest that some ligands simply do not perturb this loop geometry, whereas others do.

The overall question of the relationship of these crystal structures to the structures and complexes present in solution can only be addressed once much more extensive data are obtained. However, there is certainly sufficient data for some tentative conclusions to be drawn. One important issue concerns the loops defined here—in particular, whether or not these structures are present in solution. Importantly, there is indeed evidence for the existence of type-1 loops in solution. The NMR structure of d[TAGGG(TTAGGG)]_3_ in crowded conditions (PDB ID 2LD8) clearly shows stacked type-1 loops for all three TTA loops of all 10 structures present in the deposited ensemble (51). Thus, the type-1 loop—which is observed frequently crystallographically—appears to be highly stable and is very much present in solution. A structural alignment of the crystal and NMR structures, 1KF1 with 2LD8, (excluding the 5′ flanking residues) gives an average RMSD of 1.981 Å (see Supplementary Table S2)—this is an excellent correlation between solution and solid state. The overall visual alignment of these two structures (1KF1 and 2LD8) may be observed by scrolling through the NMR ensemble and crystal structures and is very close. Figure 2 and Table 2 also show it is common for the loops to have at least one unstacked base, which is highly likely to be adopting multiple conformations in solution. The patterns of base-base stacking seen in almost all the loop types, on the other hand, are more likely to have significant populations in solution.

An individual crystal structure provides quasi-static information about molecular structures—information embedded in temperature (B) factors in part reflects crystal lattice dynamics. Data on an ensemble of quadruplex crystal structures in which the principal variables are the bound small molecules, can, on the other hand, provide a detailed view of conformational trends in variable regions, i.e. the loops. The over-riding conclusion from this survey of available crystal structures is that these HTQ-ligand complexes have a significant degree of loop conformational variability (albeit with certain trends of conformational preference), which adds another layer of complexity to HTQ structures, and which needs to be taken into account when considering them as drug targets for *in silico* screening and molecular design studies. This feature of loops is likely to be common to all quadruplexes with loops, whether they contain single-nucleotide or much longer loops.

One key question posed by this survey is whether a particular loop conformational class is related to the nature of the bound ligand. The type-1 loop occurs at least once in each of all six naphthalene diimide complexes, in the berberine complex and in both mesoporphyrin complexes, whereas it does not occur in the salphen, or in the other porphyrin (TMPyP4) complex. Figure 3h, i shows that for the naphthalene diimide complexes, there is close correspondence between the detailed conformations of type-1 loops at the individual torsion angle level, but this is not the case for the other type-1 loops, where there is definite (albeit sometimes small) conformational variation between them. The type-1 loop also occurs in the structure of the DNA quadruplex acridine derivative (BRACO-19) complex, but not in the RNA one—one has to bear in mind that the acridines in these two structures are structurally very distinct from one another. The native RNA quadruplex shares with the two native DNA quadruplexes the preference for type-1 loops. The loop difference between the native and acridine-bound RNA quadruplexes is due mainly to the presence of the acridine, which exploits (and presumably stabilises) an extended binding surface created by the addition of loop adenine residues to the 5′ G-quartet—a (re)arrangement very much dependent on hydrogen bonds involving the *C*2’ hydroxyl groups of the ribose sugars. Overall the type-1 loop is the dominant one in the majority of these crystal structures.

Overall we conclude that loop types and conformations are most conserved within a closely-related group of small molecule complexes, as in the series of ND-quadruplex structures. In these, the differences between the structures are in the nature of the ND end-groups—all the ND compounds are tetra-substituted but with different cationic termini. There is some degree of structure conservation of type-1 loops beyond the ND complexes, especially when the ligands are not over-sized, as is the case for the acridine BRACO-19 and the berberine derivative. The implications for rational drug design of quadruplex-binding ligands are clear: *in silico* modelling based on existing crystal structures is most reliable when modelling closely similar analogues, but even then the variability in some loops is unpredictable and can best be determined by further experimental structures rather than for example, molecular dynamics with restricted simulation times. Taking one of the existing crystal structures (Table 1) as a starting-point will therefore be useful in providing qualitative guidance on optimising existing or related ligand hits, but extension for screening of structurally diverse libraries and scaffolds needs to be approached with caution.

## SUPPLEMENTARY DATA

Supplementary Data are available at NAR Online.

SUPPLEMENTARY DATA
