# Supplementary material for: Loop flexibility in human telomeric quadruplex small-molecule complexes
Source: Nucleic Acids Res. 2015 May 4;43(10):4785–99. doi: 10.1093/nar/gkv427 (PMC4446451; doi:10.1093/nar/gkv427)
Supplement: SUPPLEMENTARY DATA [file supp_43_10_4785__index.html]

Loop flexibility in human telomeric quadruplex small-molecule complexes — Loop flexibility in human telomeric quadruplex small-molecule complexes — SUPPLEMENTARY DATA 

# Loop flexibility in human telomeric quadruplex small-molecule complexes

## SUPPLEMENTARY DATA

**Files in this Data Supplement:**

- SUPPLEMENTARY DATA
