# Supplementary material for: Loop flexibility in human telomeric quadruplex small-molecule complexes
Source: Nucleic Acids Res. 2015 May 4;43(10):4785–99. doi: 10.1093/nar/gkv427 (PMC4446451; doi:10.1093/nar/gkv427)
Supplement: SUPPLEMENTARY DATA [file supp_gkv427_nar-00866-survey-d-2015-File002.doc]

**Supplementary information**

**Loop flexibility in human telomeric quadruplex small-molecule complexes.**

Gavin W. Collie, Nancy H. Campbell and Stephen Neidle

**Table S1**. Structural alignments of the G-quartets of human telomeric G-quadruplex crystal structures. In all cases the 12 guanines of the three G-quartets of each human telomeric G-quadruplex structure were aligned to the equivalent residues in PDB structure 1KF1 (native human telomeric G-quadruplex crystal structure). Alignments were preformed in PyMOL.

| **PDB Id** | **R.m.s.d. (Å)** | **No. of atoms aligned** |
| --- | --- | --- |
| 3T5E | 0.555 | 264 to 264 |
| 3SC8 | 0.896 | 264 to 264 |
| 3UYH | 0.915 | 264 to 264 |
| 4DA3 | 0.892 | 261 to 261 |
| 4DAQ | 0.886 | 261 to 261 |
| 4FXM | 0.570 | 264 to 264 |
| 4G0F | 0.577 | 264 to 264 |
| 3R6R | 0.550 | 264 to 264 |
| 3CDM (A) | 0.888 | 264 to 264 |
| 3CDM (B) | 1.083 | 264 to 264 |
| 1K8P | 0.692 | 264 to 264 |
| 3CCO | 1.095 | 264 to 264 |
| 2HRI | 1.133 | 264 to 264 |
| 3CE5 | 0.883 | 264 to 264 |
| 3QSC | 1.081 | 264 to 264 |
| 3QSF | 1.088 | 264 to 264 |
| **Average** | **0.8615** |  |


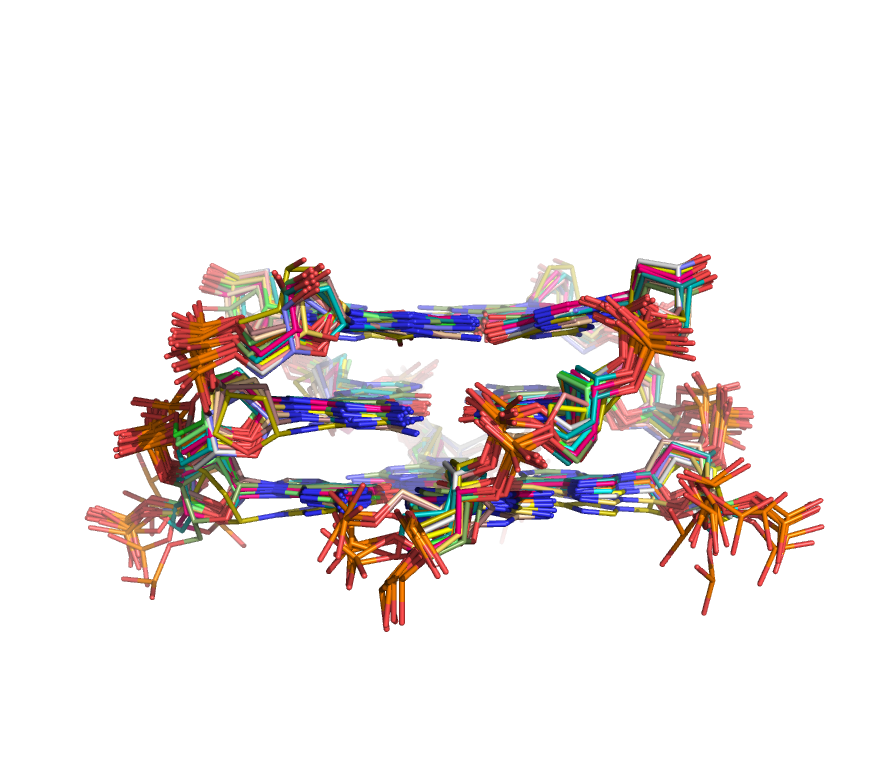

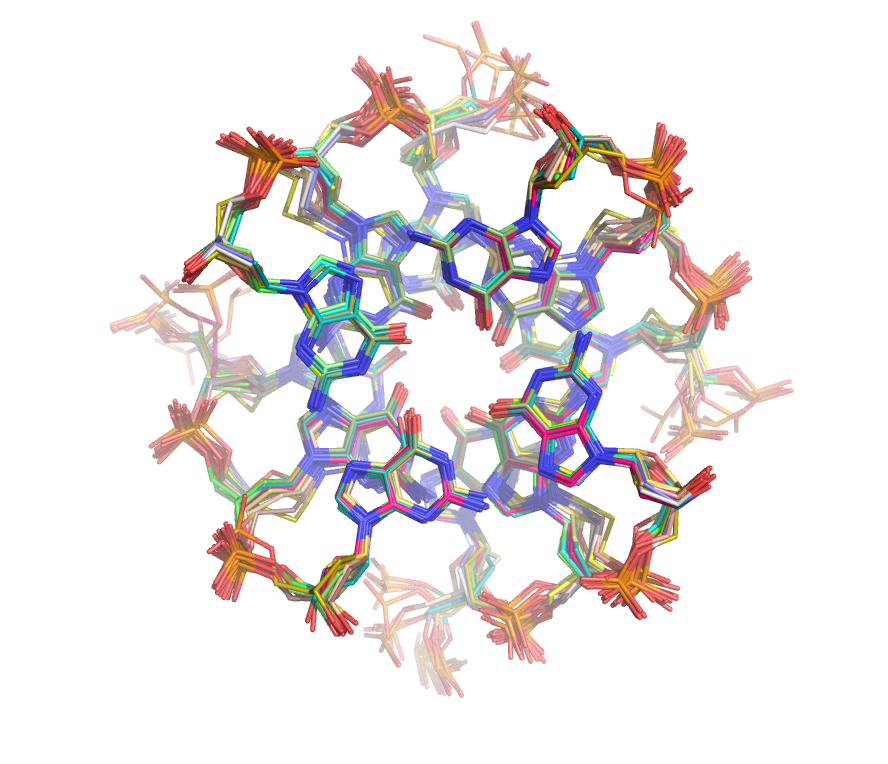


**Figure S1**. Visual representation of all structural alignments detailed in Table S1.

**Table S2**. Comparison of human telomeric G-quadruplex structures determined by crystallographic *vs* NMR methods. The crystal structure determined for the sequence AGGG(TTAGGG)_3_ (PDB id 1KF1) was structurally aligned to each of the NMR-derived models present in the ensemble of PDB entry 2LD8 (sequence: TAGGG[TTAGGG]_3_). This NMR structure was determined in ‘molecular crowding’ conditions. Alignments were performed in PyMOL, with flanking residues omitted. 447 to 447 atoms were aligned in all cases.

| **NMR model** | **R.m.s.d. (Å)** |
| --- | --- |
| 1 | 2.010 |
| 2 | 1.960 |
| 3 | 1.924 |
| 4 | 1.995 |
| 5 | 1.884 |
| 6 | 1.814 |
| 7 | 2.048 |
| 8 | 1.944 |
| 9 | 2.084 |
| 10 | 2.145 |
| **Average** | **1.9808** |
